# 

**Published:** 1911-11-15

**Authors:** 


### ANNOUNCEMENTS.

Ohio State Dental Society. The Forty-Sixth Annual Meeting of the Ohio State Dental Society will be held in the Southern Hotel, Columbus, on December 5, 6 and 7, 1911. The following program, while not giving the exact titles, indicates the subject matter of the various papers:

Dr. A. O. Ross, Columbus, President, "Most Frequent Causes of Mortality among Dentists compared with other Professions with means of Prevention."

Dr. H. T. Smith, Cincinnati, "Germicides in Dentistry."

Dr. F. W. Gethro, Chicago, "Cavity Preparation for Fillings and Inlays."

Dr. H. Printz, St. Louis, "Local Anesthetics in Dentistry."

Dr. C. R. Turner, Philadelphia, "Artistic Dental Prosthesis."

Dr. J. K. Douglas, Sandusky, "Cast Gold Inlays vs. Gold Fillings."

Dr. Henry Barnes, Cleveland, "Sweated Gold vs. Cast Gold Inlays."

Dr. L. E. Custer, Dayton, "Porcelain Inlays vs. Silicate Cement Fillings."

The above splendid program of papers, together with the usual large list of clinics, insures something of interest to every member of the profession.

Much more commodious quarters have been arranged for the sessions of the Society insuring ample room for all.

All reputable dentists are cordially invited.

F. R. Chapman, *Sec'y.*

---

The Indiana Dental Examiners will meet January 8th, 1912, in Indianapolis, in the Capitol Building, and continue in session for registration of dental practitioners for four days.

F. R. Henshaw, *Sec'y,*
Pythian Bldg., Indianapolis.

---

The Chicago Dental Society has planned for an enormous two day's celebration January 22nd and 23rd, 1912. This will be the 48th annual meeting of the society. We will have clinics on both days and papers read by the best men obtainable on both evenings.

This society has attained a membership of almost 1200 and we propose to make this one of the greatest celebrations we have ever attempted. The various officers are given on the heading of this paper and if you will insert some notice of this proposed meeting in your publication, we will be very grateful for the courtesy.

Thanking you kindly for your assistance.

Yours very sincerely,

FRED. W. GETHRO,

Chairman Publicity Committee.

---

ANNUAL MEETING. INSTITUTE OF DENTAL PEDAGOGICS. "The next annual meeting of the Institute of Dental Pedagogics will be held in Chicago, January 24th, 25th and 26th, 1912. (Notice change of dates.)

The officers and committeemen have arranged a program that will be of unusual interest to every dental teacher. In addition to the excellent papers and instructive exhibits, one day has been set apart for the inspection of the Chicago schools, where all departments will be in operation.

A detailed program will be given in the next issue of this journal."

FRED. W. GETHRO, *Sec'y.*

